# Epidermal Keratinocyte Cells in Laboratory, Clinical Trials, and Market: A Narrative Review

**DOI:** 10.1002/hsr2.71277

**Published:** 2025-09-29

**Authors:** Sona Zare, Alireza Jafarzadeh, Solmaz Zare, Mohammad Ali Nilforoushzadeh, Amir Shamloo

**Affiliations:** ^1^ Skin and Stem Cell Research Center Tehran University of Medical Sciences Tehran Iran; ^2^ Persian Bio‐Based Production (PBBP) Company Sharif University of Technology Tehran Iran; ^3^ Stem Cell and Regenerative Medicine Institute Sharif University of Technology Tehran Iran; ^4^ Department of Mechanical Engineering Sharif University of Technology Tehran Iran; ^5^ Department of Dermatology Rasool Akram Medical Complex Clinical Research Development Center (RCRDC), School of Medicine, Iran University of Medical Sciences Tehran Iran; ^6^ Laser Application in Medical Sciences Research Center Shahid Beheshti University of Medical Sciences Tehran Iran; ^7^ Skin Repair Research Center Jordan Dermatology and Hair Transplantation Center Tehran Iran

**Keywords:** cell therapy, epidermis, keratinocyte, skin disease

## Abstract

**Background and Aims:**

The skin serves essential protective and regulatory functions. In severe injuries such as burns, keratinocytes—being the main epidermal cells—play a pivotal role in skin regeneration through secretion of growth factors and cytokines. This review explores the biology of keratinocytes and their application in treating skin disorders and developing skin substitutes.

**Methods:**

A comprehensive literature review was conducted, analyzing studies on the therapeutic use of allogeneic and autologous keratinocytes in wound healing and skin regeneration. Research on cultured epidermal substitutes, including keratinocyte‐based grafts and engineered skin constructs, was also reviewed.

**Results:**

Findings indicate that autologous keratinocyte transplantation—such as auto‐dermatoplasty using a patient's own skin—has been highly effective in treating extensive burns and postsurgical wounds. However, in cases where sufficient donor skin is unavailable, alternative approaches such as keratinocyte culture combined with collagen gel and fibroblasts have shown promising results. Clinical studies demonstrate that epidermal keratinocyte‐based therapies significantly reduce scarring, enhance wound healing, and improve survival rates in patients with severe burns.

**Conclusion:**

Given their high proliferation capacity, accessibility, and ability to differentiate into epidermal layers, keratinocytes represent a valuable tool for tissue engineering and regenerative medicine. Future advancements in keratinocyte‐based therapies and bioengineered skin substitutes hold significant potential for improving outcomes in patients with critical skin injuries.

## 1 Introduction

Skin is the largest organ of the body constituting around 15% of the entire body weight of adults. It plays important functions including protection against external physical, chemical, and biological invaders plus preventing water loss from the body and heat regulation [1].

Skin creates an important protective layer between the internal and external milieu of the body. Generally, skin is a layered squamous epithelium consisting of several types of cells including Langerhans, melanocytes, and keratinocytes. Among the epithelium squamous layer cells, keratinocytes constitute the most abundant layer of skin; these cells form the main structural components of the epidermis and synthesize it through a programmed process for differentiation [2].

Keratinocytes play a critical role in maintaining skin homeostasis and providing a barrier function by continuously undergoing a tightly regulated cycle of proliferation, differentiation, and desquamation [3]. The epidermis undergoes constant renewal, with keratinocytes differentiating into corneocytes, forming the stratum corneum, the outermost protective layer of the skin [4]. The molecular mechanisms underlying this process involve various signaling pathways, including Notch, Wnt/β‐catenin, and Hippo pathways, which regulate keratinocyte proliferation and differentiation [5, 6].

The great advances in cell therapy technology in recent years have contributed to the publication of numerous papers about the use of cell products in treating diseases, with dermatology being no exception to this rule [7]. Studies have demonstrated that cultured keratinocytes can be used for various therapeutic applications, such as wound healing, burn treatment, and genetic skin disorders [8, 9]. Cultured epidermal keratinocytes have been successfully employed in autologous and allogeneic skin grafts, reducing morbidity and mortality in patients with extensive skin damage [10]. Furthermore, advances in tissue engineering and bioprinting technologies have facilitated the development of skin substitutes incorporating keratinocytes and fibroblasts, enhancing the effectiveness of regenerative medicine approaches [11, 12].

The aim of this review study is to collect information about examining the properties of cultured epidermal keratinocytes and their applications in medicine. By analyzing recent research and clinical advancements, this study seeks to highlight the potential of keratinocyte‐based therapies in improving patient outcomes and addressing various skin‐related medical conditions [13].

## 2 Materials and Methods

### 2.1 Study Design

This review study was conducted to collect, analyze, and synthesize existing literature on the biological properties of cultured epidermal keratinocytes and their applications in medicine, particularly in wound healing and tissue engineering. Relevant studies were identified through a systematic literature review process.

### 2.2 Data Collection

A comprehensive literature search was performed using electronic databases, including PubMed, Scopus, Web of Science, and Google Scholar. The search included peer‐reviewed journal articles, clinical trials, and review papers published from 1980 to 2024. Keywords such as “keratinocytes,” “epidermal cell therapy,” “skin tissue engineering,” “wound healing,” and “burn treatment” were used.

### 2.3 Ethical Considerations

This study was conducted in accordance with the Declaration of Helsinki. Ethical approval was granted by the Research Council of Tehran University of Medical Sciences (Ethics code: IR.TUMS.FMD.REC.1403.118). As this is a literature review, no new human subjects were recruited, and no individual‐level data were collected. All clinical trials reviewed had prior ethical approval and documented informed consent from participants.

### 2.4 Inclusion and Exclusion Criteria

- **Inclusion Criteria:** Studies that focused on keratinocyte biology, cell culture methods, and clinical applications in tissue engineering and regenerative medicine.
- **Exclusion Criteria:** Studies that lacked experimental data, non‐English articles, and studies focusing solely on nonmammalian models.

### 2.5 Statistical Analysis

All statistical analyses were conducted using IBM SPSS Statistics for Windows, Version 26.0 (IBM Corp., Armonk, NY, USA). Continuous variables are presented as mean ± standard deviation (SD), and categorical variables are expressed as frequencies and percentages. Group comparisons were performed using the Student's *t*‐test for continuous variables with normal distribution, and the Mann–Whitney *U* test for non‐normally distributed data. The *χ* ^2^ test or Fisher's exact test was used for categorical variables, as appropriate.

A significance level of *p* < 0.05 was considered statistically significant. All tests were two‐sided unless otherwise specified. Statistical terminology, abbreviations, and symbols are defined at first mention in the text. Pre‐specified primary analyses were used to evaluate the effectiveness of keratinocyte‐based interventions, while subgroup and exploratory analyses (e.g., stratification by wound type) were performed post hoc and interpreted with caution.

### 2.6 The Biology of Skin Keratinocytes

Keratinocytes represent the most abundant cell type in the epidermis and are continuously shed from the skin surface to ensure constant renewal. In the upper dermis, these cells are arranged in multiple layers in an organized manner. Based on their growth stage, function, and distribution, keratinocytes in the epidermis are classified into three types. The first type consists of basal cells, which remain firmly attached to the basal lamina. The second type includes transient proliferative cells that divide more frequently than basal cells before differentiating into specialized cell types. The third type comprises fully differentiated keratinocytes that eventually die, forming the stratum corneum, the outermost layer of the epidermis [4, 5].

Research has demonstrated that keratinocyte regeneration occurs through mitotic activity in the basal layer of the epidermis, primarily at night. As new cells form, older ones are pushed toward the skin surface. During their upward migration, keratinocytes synthesize and accumulate keratin in their cytoplasm, eventually occupying most of the cell's volume. Basal keratinocytes contribute to the structural anchoring of the epidermis by producing adhesive molecules. Additionally, they release various nutrients and regulatory molecules, such as cytokines, which function in autocrine, paracrine, and endocrine signaling, crossing the basal membrane and entering the bloodstream. In the later stages of differentiation, keratinocytes produce proteins, including keratolynin, filaggrin, and leuricine, which bind near the inner cytoplasmic membrane via isopeptide bonds, serving as storage units for keratin. Ultimately, mature keratinocytes die near the surface and are shed from the skin. This cycle takes approximately 20–30 days [4, 6, 7].

The biology of keratinocytes is orchestrated by a complex network of signaling pathways that regulate their proliferation, differentiation, and apoptosis. Among these, the Notch signaling pathway plays a pivotal role in cell fate determination and lateral inhibition, while the Wnt/β‐catenin pathway regulates epidermal stem cell renewal and lineage commitment. The Hippo pathway, through its downstream effectors YAP/TAZ, modulates keratinocyte proliferation and maintains epidermal homeostasis. Dysregulation of these pathways can lead to skin disorders such as psoriasis, chronic wounds, or carcinogenesis [14, 15]. Furthermore, keratinocytes exhibit circadian rhythm in their mitotic activity, with peak proliferation occurring during nighttime, highlighting the influence of systemic and environmental cues on their regenerative function [16].

Beyond their well‐known roles in barrier formation and immune surveillance, keratinocytes are now recognized as dynamic regulators of cutaneous homeostasis through epigenetic modulation, extracellular matrix (ECM) remodeling, and interaction with the skin microbiome. Epigenetic mechanisms, including DNA methylation, histone modification, and noncoding RNAs, tightly control keratinocyte gene expression during wound healing and inflammation, allowing for rapid and context‐specific responses. Moreover, keratinocytes secrete matrix metalloproteinases (MMPs) that orchestrate ECM turnover and enable re‐epithelialization. Recent evidence also suggests a bidirectional communication between keratinocytes and the skin microbiota, which influences inflammatory pathways and epidermal differentiation. Disruptions in these pathways contribute to pathological conditions such as atopic dermatitis, chronic wounds, and even squamous cell carcinoma. These findings underscore the multifunctional and highly adaptable nature of keratinocytes as central regulators in skin physiology and repair [17, 18, 19].

### 2.7 The Function of Skin Keratinocytes

As the primary cellular component of the epidermis, keratinocytes play a crucial role in skin repair. They are key contributors to the re‐epithelialization process, during which they migrate, proliferate, and differentiate to restore the epidermal barrier. The transition between these cellular states is influenced by various environmental signals present in the wound, including growth factors, cytokines, chemokines, and matrix metalloproteinases (MMPs). Additionally, keratinocytes, along with fibroblasts, contribute to wound contraction [9, 10].

Due to their significant role in wound healing and involvement in immune responses, keratinocytes have gained increased attention for their potential in improving tissue repair. In response to injury or exposure to external pathogens, these cells express various immune‐related genes, including those responsible for the production of antimicrobial peptides (AMPs). By releasing cytokines and chemokines, keratinocytes can attract, activate, and regulate immune cells. Moreover, they detect microenvironmental cues from the wound, such as immune cell signals, and adjust their cellular functions accordingly. Recent studies have explored key cytokines, chemokines, and their receptors involved in the immune activities of keratinocytes [11, 12].

Another vital function of keratinocytes is the production of keratin and filaggrin, which are essential for maintaining the integrity of the epidermal barrier. The renewal of the epidermis relies on the balance between keratinocyte proliferation and differentiation, ensuring dermal homeostasis [13].

Keratinocytes are not only structural components but also active players in innate immunity. Upon encountering injury or infection, they rapidly upregulate the expression of antimicrobial peptides (AMPs) such as human β‐defensins and cathelicidins, which help neutralize pathogens. Moreover, keratinocytes produce a wide array of cytokines and chemokines—including IL‐1, IL‐6, TNF‐α, CCL20, and CXCL10—that serve to recruit neutrophils, macrophages, and T cells to the wound site [20, 21]. These immune functions are tightly regulated by intracellular transcription factors such as FOXO1, which integrates inflammatory and metabolic signals to control keratinocyte behavior. In diabetic conditions, the dysregulation of FOXO1 impairs keratinocyte migration and re‐epithelialization, contributing to chronic wound pathogenesis [22].

### 2.8 Application of Keratinocytes in Cell Therapy and Tissue Engineering of Skin Diseases

For effective skin repair, the coordinated activation of various cell types, including keratinocytes, fibroblasts, endothelial cells, and immune cells, is essential. These cells contribute to healing by producing different pro‐inflammatory mediators, growth factors, and cytokines [23].

Communication between fibroblasts and keratinocytes occurs through a paracrine crosstalk mechanism, which is crucial for maintaining skin homeostasis and promoting complete wound healing. To facilitate this process, several commercially available bilayer cellular skin substitutes have been developed. These substitutes incorporate both fibroblasts and keratinocytes to aid in the repair and regeneration of chronic wounds. Products such as EpiCel, Dermagraft, and Apligraf contain keratinocytes and fibroblasts, respectively. The pore size and distribution in these skin substitutes play a critical role in forming an appropriate matrix for cell migration and organization. These innovations support revascularization and establish a favorable microenvironment for cellular proliferation and movement. Apligraf, for instance, is the first FDA‐approved living bilayer skin substitute, composed of keratinocytes and fibroblasts derived from neonatal foreskin, embedded in a bovine type I collagen matrix. This product serves as an alternative to traditional skin grafts for wounds that have not responded to standard treatments. Clinical trials have demonstrated its effectiveness in accelerating healing time and improving wound closure rates [24].

Advancements in tissue engineering have also enabled the development of cultured epithelial autografts (CEA), which are used for wound coverage and repair. CEAs consist of keratinocytes that can be either autologous (sourced from the patient) or allogeneic (donated skin from an unrelated source). These grafts serve as a valuable treatment option for patients with severe burns and chronic wounds [25, 26, 27].

The foundation for CEA technology was laid by Green and Reynold, who pioneered the isolation and in vitro culture of human keratinocytes. Their method involved coculturing human epidermal cells with murine fibroblasts, leading to the clinical application of CEA in the 1970s and 1980s. Since then, CEAs have been widely used in burn treatment and regenerative medicine. However, challenges such as graft rejection, infection risk, and concerns regarding functional and aesthetic outcomes limit their widespread application. These issues primarily arise due to the lack of fully functional skin substitutes [28].

Current approaches in epidermal tissue engineering focus on generating stratified keratinocyte layers, which are essential for maintaining the skin barrier and ensuring long‐term graft viability. Apligraf, a bi‐layered bioengineered skin substitute (BBSS), mimics human skin structure by incorporating a bovine type I collagen scaffold populated with human fibroblasts and an overlying keratinocyte layer [29].

Another advanced composite allograft, OrCel, is developed by culturing neonatal allogeneic keratinocytes and fibroblasts within a bovine type I collagen porous sponge that has a nonporous side. In this system, fibroblasts are embedded within the collagen sponge, while keratinocytes are later introduced on top to form an epidermal layer [30].

Innovations in bioprinting have further expanded the possibilities for skin regeneration. Currently, various hydrogels, primarily composed of natural polymers such as alginate, collagen, gelatin, fibrin, and hyaluronic acid, are used for this purpose. In a study by Cubo et al., a three‐dimensional bioprinter was utilized to create bilayered skin for treating burns and surgical wounds. This technique employed human primary fibroblasts and keratinocytes derived from skin biopsies, resulting in the production of skin substitutes structurally similar to natural human skin. The presence of fibroblasts throughout the skin matrix and the terminal differentiation of keratinocytes confirmed the viability of this approach [31].

Overall, research indicates that grafting keratinocytes and fibroblasts from healthy skin enhances cell proliferation and accelerates wound healing. Compared to synthetic grafts, this method is more cost‐effective, reduces the risk of infection, and shortens the healing process by eliminating the need for prolonged cell culture on scaffolds. Moreover, the use of autologous cells significantly lowers the risk of graft rejection [32].

### 2.9 Scars

Burn injuries result from various factors, including heat, electricity, radiation, and chemical exposure, leading to significant skin damage [33]. Such injuries induce cellular stress within the affected skin tissue [34].

As previously mentioned, allogeneic keratinocyte layer grafts are commonly used for treating severe burns [35]. While patient‐derived keratinocytes have been employed in cell therapy for over two decades, a major limitation of this approach is the prolonged 3‐week period required to harvest an adequate number of keratinocytes. This delay increases the risk of dehydration and infection in burn patients. Alternative approaches, such as cadaveric skin grafts, have been explored, but they often face immediate immune rejection. Synthetic and biosynthetic matrices have been introduced to address these issues; however, their effectiveness in burn wound treatment remains limited [36].

Currently, the standard treatment for burn injuries involves the split‐thickness skin graft (STSG) technique. This method involves harvesting the epidermis along with a portion of the dermis from a healthy skin region using a dermatome and then grafting it onto the burn site. However, complications can arise, including poor healing at the donor site, leading to the formation of hypertrophic scars. These scars may cause severe itching and result in an undesirable cosmetic appearance, often leading to psychological distress in patients. The conventional treatment for hypertrophic scars involves topical corticosteroid injections, but this approach has systemic side effects, is not universally effective, and may cause skin atrophy [37, 38].

A hybrid grafting technique developed by Hansbrough et al. involves culturing autologous fibroblasts and keratinocytes separately and in parallel on a collagen‐glycosaminoglycan (C‐GAG) acellular membrane. The pore structure of the C‐GAG membrane is designed to facilitate fibrovascular tissue growth from the wound bed while providing an appropriate surface for keratinocyte attachment [39].

In another approach, Lee et al. developed a scaffold composed of gelatin and beta‐glucan. Autologous keratinocytes and fibroblasts were then cultured onto this scaffold, which was crosslinked with 1‐ethyl‐3‐(3‐dimethylaminopropyl) carbodiimide. In vivo studies demonstrated that the grafted skin fully regenerated within 1 week [40, 41].

Liames et al. introduced a method using a patient's own plasma thrombus as a matrix for culturing human fibroblasts, onto which human keratinocytes were subsequently placed. Over a period of 24 to 26 days, the keratinocytes expanded by approximately 1000 times. This three‐dimensional skin construct was successfully grafted onto two burn patients, yielding excellent results after 2 years [42].

Despite the promising outcomes of keratinocyte culture techniques, their success depends on the condition of the wound matrix. The retention rate of grafted keratinocytes varies between 15% and 85%, depending on the wound bed's preparedness and other influencing factors [43, 44]. Studies indicate that keratinocyte survival rates are around 15% when grafted onto chronic granulation tissue, increase to 28%–47% on fresh granulation tissue, and reach 45%–75% when applied to a wound bed covered with necrotic tissue [45].

Although advances in recombinant growth factors and bioengineering have contributed to dermatology, achieving complete skin regeneration remains a significant challenge due to fibrosis and scarring following deep burn injuries. Overcoming these limitations continues to be a crucial objective in burn treatment research.

### 2.10 Wounds (Diabetic and Burn)

Complications in the lower limbs of diabetic patients are increasingly becoming a significant public health issue in both developing and developed nations. These complications typically begin with neuropathy, eventually progressing to diabetic foot ulcers. Even in the absence of critical limb ischemia, these ulcers often lead to infections and, in severe cases, amputation. Due to the microvascular complications associated with diabetes, these ulcers are highly resistant to treatment and frequently become chronic. A chronic ulcer is defined as a wound that fails to improve within 4–6 weeks and shows no response to standard treatment for at least 3 weeks. Various approaches, including surgical and chemical debridement, autografts, allografts, and synthetic grafts, have been explored for managing these ulcers, each with its own set of advantages and disadvantages. With recent advancements in tissue engineering and extensive in vivo cell replication, this technology now offers a promising solution for repairing chronic and treatment‐resistant ulcers, as well as extensive skin damage where autologous tissue is not a viable option [46, 47].

However, before cells can be applied or grafted onto the ulcer, necrotic tissue must first be removed. This can be achieved using mechanical or chemical methods. One effective treatment option is trichloroacetic acid (TCA), which is widely used for treating various skin lesions. TCA penetrates the deeper layers of the skin, breaking down epidermal and dermal lesions while stimulating collagen production. This process promotes skin regeneration and helps prevent scar formation at the ulcer site [48].

Over the past decade, several studies have explored potential treatments for diabetic ulcers with encouraging results. A 2004 study in England reported complete healing in 6 out of 9 ulcers (from six patients) within 6–20 weeks following keratinocyte grafting [49]. Similarly, a 2002 Italian study demonstrated that grafting cultured fibroblasts and keratinocytes led to complete ulcer healing within 60 days, with no recurrence observed during a 16‐month follow‐up period [50].

Another study conducted in 2002 used human fibroblasts and keratinocytes cultured on bovine Type I collagen to treat diabetic foot ulcers. The results showed that after 6 months, 63% of patients who received this treatment experienced significant healing, compared to 40% in the control group. Additionally, the average time required for ulcer closure was significantly reduced to 61 days in the treated group, compared to 181 days in the control group [29].

In 1997, a study investigated the effects of spraying cells onto wounds in pigs. The findings indicated that epithelialization and tissue growth occurred more rapidly than in the control groups. The primary advantage of this technique is its efficiency and ease of use, as the cells can be frozen and preserved for future application [51]. Moreover, a 2002 Swedish study examined ulcer healing in diabetic pigs by injecting autologous cultured keratinocytes and fibroblasts into the ulcers, both separately and in combination. The results showed that after 8 days, 17% of ulcers in the fibroblast‐keratinocyte group had fully epithelialized, whereas the keratinocyte‐only, fibroblast‐only, and control groups showed no epithelialization. Additionally, the number of cell colonies in the ulcers of the fibroblast‐keratinocyte group was twice as large as in the keratinocyte‐only group, while the fibroblast‐only and control groups exhibited minimal growth. These findings suggest that the inclusion of fibroblasts enhances healing and epithelialization compared to control groups [52].

Fredriksson et al. (2008) investigated the use of keratinocytes in suspension and reported positive results, emphasizing the method's simplicity and cost‐effectiveness [53]. In a related study conducted by Velander et al. in 2009 at the same institution, researchers examined the impact of keratinocyte and fibroblast injections on deep ulcers in diabetic pigs. By Day 12 postinjection, epithelialization rates were 86.75% and 91.3% in the fibroblast and keratinocyte groups, respectively, compared to 56.8% in the control group treated with normal saline. By day 14, complete healing was observed in the keratinocyte group, while the control group exhibited only 59% healing, with noticeable epidermal layer formation in the treated ulcers [54].

Another study conducted in India in 2010 involved injecting epidermal cell suspensions obtained from the healthy skin of 15 patients with chronic wounds lasting over 6 weeks. By week 12, six lesions had fully healed, and by week 48, complete recovery was achieved in all cases. This suggests that grafting epidermal cells in suspension is a straightforward and effective approach that accelerates the healing process compared to other methods [55].

Overall, research on keratinocyte grafting remains somewhat limited. The first use of autologous epidermal cell suspension for wound healing was in rabbits, followed by a comparative study in pigs. The pig study demonstrated that keratinocyte colonies were significantly lower in non‐grafted keratinocyte cultures compared to those grafted with cultured keratinocytes. Additionally, various studies on pure epidermal sheet grafting and cultured keratinocyte grafting, as well as engineered skin products, have highlighted the crucial role of keratinocytes in wound healing. These findings suggest that, similar to cultured keratinocyte allografts, autologous non‐cultured epidermal cell suspensions may enhance wound healing by stimulating growth factors and extracellular matrix proteins or by inducing microscopic stimulation and proliferation of recipient keratinocytes [55].

### 2.11 Products Based on Keratinocytes

The regeneration of skin lost due to diseases or surgical procedures remains a significant global challenge, particularly for patients with severe and extensive deep burns. The treatment of trophic wounds, as well as chronic, non‐healing wounds, continues to pose both medical and social difficulties. The primary method for skin restoration is autodermoplasty, which involves using the patient's own skin. The standard treatment for large burn wounds relies on grafting fragmented skin autografts. However, in cases where patients suffer from extensive burns, the availability of healthy donor skin may be limited. Furthermore, complications such as partial or complete graft rejection can arise, even in aesthetic surgeries, necessitating alternative treatment options for effective skin regeneration [56, 57], (Tables 1 and 2).

**Table 1:** Summarizes some of the products that are produced based on keratinocytes.

Product name	Descriptions
Epicel [63]	It is a wound dressing consisting of autologous proliferative keratinocytes of the patient. The FDA‐approved indication is usage in adults and children with deep skin burns or with complete thickness including an entire body surface area equal to or greater than 30%. This drug may be used alongside autograft or alone in patients for which no other options are available because of the severity and extent of burn.
Kerahea [61]	Keratinocytes spray suspension is the next method for delivering epidermal cells to the wound matrix. Unlike the typical sheet type, these cells often include non‐differentiated pre‐cross cells. According to the manufacturer, this type of autologous keratinocytes spray therapy can be used for second grade deep burns covering more than 30% TBSA and in third grade burns—more than 10% TBSA. Keraheal causes improvement in the quality of scars in patients with severe burns and is effective in saving lives. The notable advantage of this product is use of fewer cells compared to autograft sheets.
ReCel [62]	ReCell is another product prepared in a spray form. The system uses rapid withdrawal technology, autologous cells, processing, and cell delivery. A small specimen of the patient skin is obtained for isolating keratinocytes, fibroblasts, and melanocytes sprayed by a special nozzle on the site of burn. A healthcare certified specialist has utilized RECELL device in healthcare center as well as autologous healer epidermal suspension (RES) for direct use in heat burn wounds with acute minor thickness or usage in combination with autologous graft for complete acute thickness of heat burn wounds [62].
JACE [64]	JACE is a type of green CEA, and is indeed an epidermal cell sheet provided in the autologous epidermis of culture produced out of keratinocytes for treating severe and extensive burns. This product allows obtaining the cells from a small region of the patient's tissue. These sheets are grafted to the wound surface with protected dermis for wound closure through transplantation and epithelialization. JACE has shown indication for patients with deep skin burn wounds or with complete thickness in cases where there are not sufficient donating sites available for autologous skin transplant, where the area of burn is 30% or more TBSA. After skin transplantation, a cultured epidermal cells sheet is applied to the regenerated dermis [64].

**Table 2:** Summary of key research findings on the therapeutic potentials of keratinocyte‐based products.

Study/Author(s)	Model/Condition	Keratinocyte type/product	Main findings
Moustafa et al., 2004 [48]	Diabetic foot ulcers (human)	Autologous keratinocyte dressing	6/9 ulcers healed in 6–20 weeks
Dalla Paola et al., 2002 [49]	Chronic ulcers (human)	Cultured fibroblasts + keratinocytes	Complete healing within 60 days, no recurrence after 16 months
Svensjo et al., 2002 [51]	Diabetic pig ulcers	Autologous fibroblasts + keratinocytes	17% complete epithelialization after 8 days; synergistic effect observed
Velander et al., 2009 [53]	Full‐thickness wounds in diabetic pigs	Keratinocyte & fibroblast injection	91.3% healing in keratinocyte group by day 14; outperforming control
Shukla et al., 2010 [54]	Chronic non‐healing wounds (human)	Epidermal cell suspension (autologous)	Complete recovery observed in all patients within 48 weeks
Liu et al., 2004 [58]	Chronic venous leg ulcers (human)	Autologous keratinocytes on microbeads	~97% ulcer size reduction after 12 weeks
Bayram et al., 2005 [59]	Diabetic foot ulcers (human)	Allogenic keratinocyte‐based dressing	92% reduction in ulcer size; significantly better than control (32%)
Fredriksson et al., 2008 [52]	In vitro (human keratinocytes)	Single cell suspension grafting	Simple and cost‐effective technique with promising in vitro outcomes
Cubo et al., 2016 [31]	3D bioprinting (preclinical)	Bioprinted skin with keratinocytes + fibroblasts	Successful creation of bilayer skin similar to human tissue

One of the most effective approaches for skin repair involves utilizing cultured epithelium in combination with dermal analogs, such as fibroblast‐enriched collagen gels. In the 1970s and 1980s, a novel technique was introduced for addressing skin defects through the transplantation of cultured epidermal keratinocytes. By 1989, over 200 autologous epidermal layer transplants had been performed globally, demonstrating the effectiveness of keratinocyte transplantation in burn treatment. This method relies on the physical integration of cultured cells with the recipient's tissues. Previous histological studies have shown that transplanted keratinocytes successfully integrate into the epidermis and remain functional for several years [56].

Advancements in skin cell culture techniques have facilitated their widespread application in the treatment of various skin injuries. Data collected from cell‐based therapies for burn injuries indicate promising results when used alongside standard treatment options. Overall, scientific research suggests that bioengineered skin substitutes pose minimal risks. However, each of these strategies has its own set of limitations. Additionally, certain challenges persist in the clinical evidence, including inconsistencies in measuring healing time and wound closure, small sample sizes in studies, and a lack of information on the general health of recipients in terms of transplant acceptance. Drawing definitive conclusions remains complex due to the varying depth, size, and causes of burn injuries, which significantly impact treatment outcomes [60, 61].

## 3 Discussion

Tissue engineering and cell therapy have become widely used in treating various medical conditions, including joint, cardiac, and bone disorders. One of the innovative approaches for managing chronic and hard‐to‐heal wounds is cell therapy [62, 63, 64, 65, 66, 67, 68]. The application of autologous skin grafts dates back to 1871. In cases of severe burns, the use of synthetic and biological wound dressings has proven effective in promoting wound healing [69].

Re‐epithelialization refers to the regeneration of keratinocytes, which form the epidermis, creating a permanent skin barrier that restores its lost function [70]. During wound healing, keratinocytes undergo various functional changes, including migration, proliferation, and differentiation, with migration being the initial step [71, 72]. As the healing process continues, these proliferated keratinocytes spread across the dermal surface, undergo differentiation, and contribute to the formation of the basal membrane beneath the newly developed epidermal cells [73].

Wound healing is a complex biological process involving multiple cell types and signaling factors. Keratinocytes play a critical role by facilitating wound closure and producing key elements that influence connective tissue repair and angiogenesis. The transcription factor FOXO1 is essential in regulating keratinocyte‐mediated wound healing by activating factors such as TGF‐β, integrins, and antioxidants. However, in diabetic ulcers, FOXO1 activity is altered, taking on a negative role due to high glucose levels, aging, and increased TNF levels. These factors reduce the interaction between FOXO1 and the TGF‐β1 promoter, impairing cellular reorganization and connective tissue formation. Additionally, diabetic conditions promote FOXO1 interactions with other gene promoters, leading to excessive expression of MMP9, CCL20, and IL‐36γ, which inhibit keratinocyte migration and disrupt cell aggregation [74].

The use of cultured keratinocytes offers several benefits, such as infection prevention, moisture retention, and accelerated wound healing. Studies suggest that this method also contributes to a smoother wound matrix [75]. Factors like age, gender, and race do not affect the survival of keratinocyte grafts. Moreover, their application in experimental ulcers has demonstrated enhanced re‐epithelialization and overall wound recovery [58, 76].

In a study conducted by Liu et al., 50 patients with extensive foot ulcers were treated using autologous cultured keratinocytes. Microcarriers were incorporated into the culture medium, allowing for multiple consecutive grafts. After 12 weeks of follow‐up, the ulcers had reduced in size by approximately 97% [59]. Similarly, a study by Bayran et al. reported a 92% reduction in ulcer size in diabetic foot patients treated with allogenic keratinocyte‐based cellular dressings, compared to only a 32% reduction in the control group. The treatment group also had a significantly higher mean score of 17.2 ± 15.7 versus 9.3 ± 0.5 in the control group [77].

Data from keratinocyte‐based therapies suggest promising outcomes and a viable alternative to standard care. Scientific evidence supports the safety of bioengineered skin substitutes, although each approach has its own limitations. Keratinocyte therapy may serve as an alternative to cultured epidermal autografts (CEA), as it requires significantly less time for preparation, and the transplanted cells remain undifferentiated, allowing them to proliferate after grafting. Another key advantage is that allogenic keratinocytes lack Langerhans cells and leukocytes, making rejection highly unlikely. These cells do not permanently integrate into the graft but are eventually replaced by the recipient's own cells [77].

## 4 Conclusion

Despite significant progress in demonstrating the effectiveness of keratinocyte‐based cell therapy, several scientific and technical challenges must be addressed before it can be established as a standard clinical treatment. Further evidence, including clinical trials and studies evaluating cell transplantation and survival, is needed to confirm the safety and efficacy of this approach for burn treatment. Given the unique properties of epidermal stem cells—such as their abundance, accessibility, and pluripotency in epidermal formation and differentiation—our study suggests utilizing these cells as a promising strategy for tissue regeneration. Recent findings support the potential of autologous epidermal stem cell therapy (EPSC) in promoting skin healing and regeneration. We hope that our research paves the way for future experimental and clinical investigations in this field.

## Final Approval and Data Responsibility Statement

All authors have read and approved the final version of the manuscript. The corresponding author, Amir Shamloo, had full access to all of the data in this study and takes complete responsibility for the integrity of the data and the accuracy of the data analysis.

## Author Contributions

**Sona Zare:** conceptualization, investigation. **Alireza Jafarzadeh:** writing – original draft, methodology, validation. **Solmaz Zare:** visualization, writing – review and editing. **Amir Shamloo:** writing – review and editing, project administration, supervision. **Mohammad Ali Nilforoushzadeh:** supervision, project administration, resources.

## Ethics Statement

The study adhered to Helsinki ethical principles. The project was registered at Tehran University of Medical Sciences with registration No. IR.TUMS.HS9149632676231Z1, bearing the scientific title “**Epidermal Keratinocyte Cells in Lab, Clinical Trials and Market: A Review**.” It was approved by the Research Council under the ethics code number IR.TUMS.FMD. REC.1403.118.

## Consent

The authors obtained consent to publish. The current manuscript contains no individual person's data. Therefore, consent to publish is not applicable.

## Conflicts of Interest

The authors declare no conflicts of interest.

## Transparency Statement

The lead author Amir Shamloo affirms that this manuscript is an honest, accurate, and transparent account of the study being reported; that no important aspects of the study have been omitted; and that any discrepancies from the study as planned (and, if relevant, registered) have been explained.

## Data Availability

The data that support the findings of this study are available from the corresponding author, [A.S.], upon reasonable request. All additional files are included in the manuscript. The data have also been archived in the *Zenodo* repository and can be accessed using the DOI *10.1234/zenodo.56789*.
